# Modeling the environmental suitability for *Bacillus anthracis* in the Qinghai Lake Basin, China

**DOI:** 10.1371/journal.pone.0275261

**Published:** 2022-10-14

**Authors:** Temitope Emmanuel Arotolu, HaoNing Wang, JiaNing Lv, Kun Shi, Hein van Gils, LiYa Huang, XiaoLong Wang

**Affiliations:** 1 Center of Conservation Medicine & Ecological Safety, Northeast Forestry University, Harbin, Heilongjiang Province, P. R. China; 2 Key Laboratory of Wildlife Diseases and Biosecurity Management, Harbin, Heilongjiang Province, P. R. China; 3 College of Wildlife and Protected Area, Northeast Forestry University, Harbin, Heilongjiang Province, P. R. China; 4 School of Geography and Tourism, Harbin University, Harbin, Heilongjiang Province, P. R. China; 5 Wildlife Institute, Beijing Forestry University, Beijing, Beijing, P. R. China; 6 Changbai Mountain Academy of Sciences, Antu, Jilin Province, P. R. China; USGS WCWRU: US Geological Survey Wisconsin Cooperative Wildlife Research Unit, UNITED STATES

## Abstract

*Bacillus anthracis* is a gram-positive, rod-shaped and endospore-forming bacterium that causes anthrax, a deadly disease to livestock and, occasionally, to humans. The spores are extremely hardy and may remain viable for many years in soil. Previous studies have identified East Qinghai and neighbouring Gansu in northwest China as a potential source of anthrax infection. This study was carried out to identify conditions and areas in the Qinghai Lake basin that are environmentally suitable for *B*. *anthracis* distribution. Anthrax occurrence data from 2005–2016 and environmental variables were spatially modeled by a maximum entropy algorithm to evaluate the contribution of the variables to the distribution of *B*. *anthracis*. Principal Component Analysis and Variance Inflation Analysis were adopted to limit the number of environmental variables and minimize multicollinearity. Model performance was evaluated using AUC (area under the curve) ROC (receiver operating characteristics) curves. The three variables that contributed most to the suitability model for *B*. *anthracis* are a relatively high annual mean temperature of -2 to 0°C, (53%), soil type classified as; cambisols and kastanozems (35%), and a high human population density of 40 individuals per km^2^ (12%). The resulting distribution map identifies the permanently inhabited rim of the Qinghai Lake as highly suitable for *B*. *anthracis*. Our environmental suitability map and the identified variables provide the nature reserve managers and animal health authorities readily available information to devise both surveillance strategy and control strategy (administration of vaccine to livestock) in *B*. *anthracis* suitable regions to abate future epidemics.

## Introduction

Anthrax is an infectious, often fatal disease of wild and domestic animals and humans that is caused by the endospore-forming, soil-borne and gram-positive *Bacillus anthracis*. It is primarily a disease found in herbivores but direct or indirect contact with contaminated animals can lead to outbreaks in humans, with potentially serious consequences’ [1]. Herbivorous mammals are infected when grazing on contaminated grass, bitten by tabanid flies with contaminated mouthparts or ingesting contaminated carcasses [1]. Herders, livestock farmers, workers in abattoirs, meat and fur processing plants and veterinarians are exposed to the disease as an occupational hazard. Recently, the disease has transposed from industry to agriculture affecting farmers and herdsmen in 87.6% of the human cases in China [2]. *B*. *anthracis*, the etiological agent of anthrax, exhibits a bimodal lifestyle consisting of the vegetative and the spore stage [3]. Bacteria in the vegetative stage are shed by infected animals and may die rapidly in most environmental conditions. After sporulation from the vegetative cells, the *B*. *anthracis* can survive in the soil for decades [4]. The bacillus replicates rapidly in the bloodstream to high concentrations and releases toxins resulting in septicemia, which soon kills the susceptible host. In soil and vegetation, the spore can remain viable and infectious for years until it comes in contact with and enters a new susceptible host where it germinates and begin a new life cycle [5]. Human anthrax infections are caused by contact with infected animals or animal products, ingestion of undercooked infected meat; or exposure to processing of contaminated hides, wool, and hair in enclosed spaces [6].

Clinically, there are three forms of anthrax namely; cutaneous, gastrointestinal tract and pulmonary (inhalation). Globally, cutaneous anthrax accounts for over 95% of the human cases with 97.7% recently reported in China [2] and are rare in livestock and wildlife. Due to *B*. *anthracis’* virulence, tenacious anthrax cases and repetitive outbreaks, concerns have been raised across continents in recent years, e.g., sub-Saharan Africa [7], Asia [8], Europe [9], Australia [10], and the North America [11]. Also due to its potential use for bioterrorism, anthrax is considered as a global public health threat [12]. Effective vaccines in livestock have reduced the economic significance of the disease in developed countries where it now occurs sporadically in unvaccinated domestic stock and wildlife populations [13]. In the Qinghai Province of China, anthrax occurs sporadically and all year round. The prevalence of anthrax in Qinghai rose from 0.35/100,000 in 2012 to 1.17/100,000 in 2016. The incidence is gradually increasing as well [14]. Qinghai province has recently been identified as one of the potential sources of *B*. *anthracis* in a recent study characterizing the distributional patterns of both human and livestock anthrax in China [15, 16]. The study used both clinically and laboratory-confirmed cases during 2005–2013, routine surveillance of livestock anthrax was conducted by the Ministry of Agriculture of the People’s Republic of China [15].

Anthrax in livestock has been controlled, but not in wildlife. The behavior of avian and mammalian scavengers and alternative routes (waterborne transmission and flies) has proved unimportant relative to the long-term persistence of anthrax spores in soil and their infection of herbivore hosts [17]. However, human beings, livestock, and wildlife will invariably encounter each other or share habitats and other resources at the interface areas which would cause a spill back or spillover of the infection. Livestock vaccination and intensive surveillance of disease are essential for anthrax prevention [18]. However, widespread surveillance is costly, therefore, there is a need to concentrate intensive control measures in high-risk areas by increasing the understanding of *B*. *anthracis* ecology.

Similar studies in China have identified climatic variables and human population density as good predictors of *B*. *anthracis* suitability [15]. Presence-only modeling algorithms to predict the environmental suitability of *B*. *anthracis* have been widely used, including maximum entropy [19] and GARP (genetic algorithm for the rule-set prediction) [10]. During comparative model studies, MaxEnt outperformed other algorithms [20]. We tested the hypothesis that soil type, climatic variables and human population density are significant predictors of *B*. *anthracis* suitability in the Qinghai Lake basin. We predicted the distribution of *B*. *anthracis* in Qinghai Lake basin using MaxEnt algorithm [21, 22]. This study revealed the environmental suitability areas and variables responsible for *B*. *anthracis* distribution, which could help local authorities to devise both surveillance and control strategy to forestall future outbreak of anthrax in Qinghai Lake basin.

## Materials and methods

### Study area

**Fig 1:**
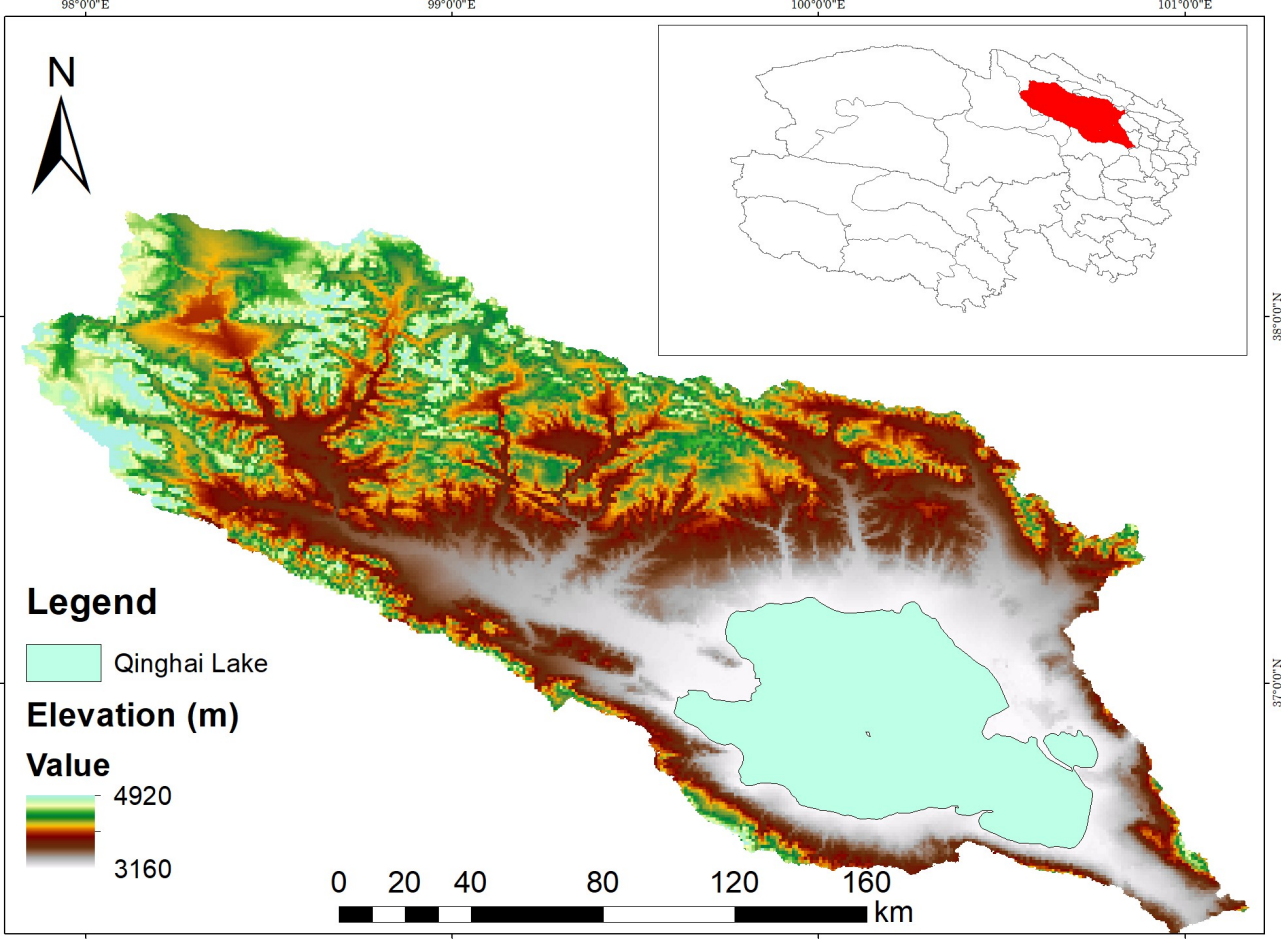
Area of Interest (AOI): Qinghai Lake basin in Qinghai province, People’s Republic of China. ArcGIS was used to process the raster obtained from WorldClim (http://worldclim.org/version2) to draw AOI map, with kind permission of Dr. Stephen Fick, geo-spatial data scientist. The boundary lines should not be reused or misinterpreted for any political reason.

Our area of interest (AOI) is the basin of the Qinghai Lake (98°37’ - 101° 45’ E and 36° 33’ - 39° 14’ N) in Qinghai province, Northwest China (Fig 1). The basin is approximately 29,600 km^2^, and the lake about 4,300 km^2^. The water surface is roughly situated at 3,193 meter above the sea level (m a.s.l.), with an average depth of 21m [23]. Qinghai Lake is situated in a closed-basin (29,661 km^2^) with no surface water outflow. The entire watershed is in a high-altitude, cold and semiarid climate zone [24]. More than 40 rivers flow into the Qinghai Lake, but most are intermittent. Qinghai Lake is the largest salt lake in China, an international wetland [25] and a breeding ground for migratory water fowl. Further, the mountains around the Qinghai Lake are perhaps the last refuge of the endangered Przewalski’s gazelle (*Procapra przewalskii*) [26]. The Qinghai Lake basin has been identified as a modern, highly efficient animal husbandry production area where human beings and nature live in harmony [27]. The human population in the entire watershed is about 110,000, mainly living around Qinghai Lake [28]. The mainstay of the rural economy in Qinghai province in China, including the lake basin is livestock husbandry [29]. Livestock mainly includes sheep, goat and yak, but also some horse, cattle and donkey. Livestock numbers per household varied from dozens to more than 1,000 [30]. In spring or early summer most livestock are transferred to high-altitude pastures (4850 to 4950 m.a.s.l) where milk is processed and herds gain weight. After returning to the homestead (3190 to 3300 m a.s.l.) in late summer, fodder (oats) and crop residues are provided as principal feed in addition to stubble grazing and grassy patches near the winter residence [31]. Grassland is the major land cover, accounting for about 63% of the AOI [27]. The main vegetation types are: needleaved forest in cold temperate, shrubs in plateau valley, alpine shrubs, sandy shrubs, steppe in temperate, alpine steppe, alpine meadow, swamp meadow, subnival vegetation and so on [32]. Two predominantly grown crops are the oilseed rape and highland barley [33].

### Anthrax occurrence data and preprocessing

We collected 37 cases of anthrax in human beings (n = 08), livestock (cattle and sheep) (n = 20) and wildlife carcasses (n = 05) from spatial records provided by the World Organization for Animal Health [34] and publications [14–16] The spatial autocorrelation was minimized by filtering all recorded anthrax locations using SDM Toolbox v1.1c in ArcGIS 10.3 [35]. Filtering was performed by limiting the minimum distance between each pair of points. In addition, the filtering program plays the role of systematic sampling. It can delete adjacent records to reduce spatial aggregation, which is regarded as the most effective method in correcting sampling bias [36, 37].

### Environmental variables and preprocessing

A total of 68 climatic, 12 incoming solar radiation (ISR) and 8 soil variables were used in our analysis (S1 Table). We extracted the climatic variables from WorldClim version 2.1 for data from 1970–2000 at 30 arc–second resolution [38, 39]. The categorical variable soil type and continuous soil variables in 1km grids were extracted from soil grids database [40] including soil organic carbon, clay, silt and sand content as well as cation exchange capacity, soil pH, and land cover/use (S2 Table). Livestock (cattle, sheep and goat) population animals/km^2^ was obtained from https://livestockdata.org/contributor/gridded-livestock-world-glw3 [41]. In addition, the human population density from the Asia Continental Population Datasets (2000–2020), which are publicly and freely available both through the WorldPop Dataverse Repository and the WorldPop project website (http://www.worldpop.org.uk/data/), were used as predictor variables in this research. Principal component analysis (PCA) was used to reduce the number of continuous environmental variables [37, 42, 43]. During PCA, we used eigenvalues larger than 0.97 and the scree plot criterion for PCA in item level factoring [44]. Suppression of unnecessary loading and rotation of factor pattern of climatic variables [45] were used to retain climatic variables. After variable reduction in PCA, we used VIF (linear regression statistics) in SPSS 22.0 to assess multicollinearity among both the remaining continuous variables and the categorical variables [46]. A VIF >10 was considered to indicate highly correlated variables, which were thus removed from the input data set. Subsequently, only seven uncorrelated variables were used (S3 Table). The Jackknife test, backward stepwise variable elimination, and the variable response curves were selected to identify the relative contribution of predictor variables to the model [22].

### Model development and evaluation

A MaxEnt model v3.4.1 was fitted using 100 bootstrap runs, with a 70/30 partition percentage for the training/testing datasets. The advanced options in MaxEnt that were selected include the maximum iteration set to 5000 to allow the models to have enough time to reach convergence at 0.00001 [47] 90% sensitivity was set within the MaxEnt model for determining suitability. The area under the Receiver Operating Characteristics [48] was used to assess the accuracy of the model. In the MaxEnt model, the Area Under the Curve (AUC) of the receiver operating characteristic plot was used as an evaluation criterion to assess the accuracy of the model [49]. The stepwise elimination approach was used to remove variables that contributed less than ten percent (10%) to the model [37]. Further, a smooth response curve was used as a quality standard [22]. We reclassified the MaxEnt spatial model output into two environmental suitability classes, namely high and low in ArcGIS v10.3.

## Results

The filtering selected 25 out of 37 presence records at 10 km rarefying. The PCA delivered five PCs, together accounting for 98.7% of the total variance (Table 1, S1 Fig). After exclusion of unnecessary factor loading, thirteen predictor variables were retained. No multicollinearity was detected with VIF values of 0 to 2 (<10) between predictors. For model validation, AUC value of 0.93, SD = 0.024 indicating that it had excellent ability to predict the suitability areas for *B*. *anthracis* (S2 Fig).

**Table 1:** Ranking of principal components by eigenvalues.

PC Rank	Eigenvalues		
	Total	Variance %	Cumulative %
1	42	63	63
2	16	24	87
3	4	6	93
4	2	3	96
5	1	2	98

Three variables contributed >10% (Table 2) namely, annual mean temperature (53%), soil type (35%), and human population density (12%). The Jackknife test of variables shows that omitting any of these three variables affects the regularization gain, test gain and AUC in the model. The annual mean temperature has the highest training gain when each variable was tested as the only environmental variable (1.2), and the lowest values were observed when analyzing only human population density (0.4). The lowest training gain appeared when the annual mean temperature was excluded from the model, while the model has the highest gain when human population density (1.2) and soil type (1.3) were excluded (Table 3).

**Table 2:** Contribution of the three environmental predictors to the final suitability model.

Variable	Contribution (%)	Permutation (%)
Annual mean temperature	53	83
Soil type	35	4
Human population density	12	13

**Table 3:** Summary of the Jackknife analysis performed to determine importance per environmental variable.

Variable	Regularized Training gain		Test gain		Test AUC	
	Alone	Excluded	Alone	Excluded	Alone	Excluded
Annual mean temperature	1.2	0.8	1.2	0.8	0.86	0.87
Soil type	0.6	1.3	0.4	1.5	0.76	0.91
Human population density	0.4	1.2	0.8	1.0	0.89	0.83

Annual mean temperature has the highest test gain values when used as the only environmental variable and soil type has the least test gain among the variables. Our model has a high training gain value when human population density and soil type were simultaneously excluded from our modeling process. The exclusion of annual mean temperature variable from the model results in a decline of the test gain (Table 3).

Observing our Jackknife test for AUC, the three important variables (annual mean temperature, soil type and human population density), when used in isolation were not significant different from each other. The AUC value of our model was excellent when soil type was excluded. There were no significantly differences in AUC values of the other two variables in the model (Table 3).

**Fig 2:**
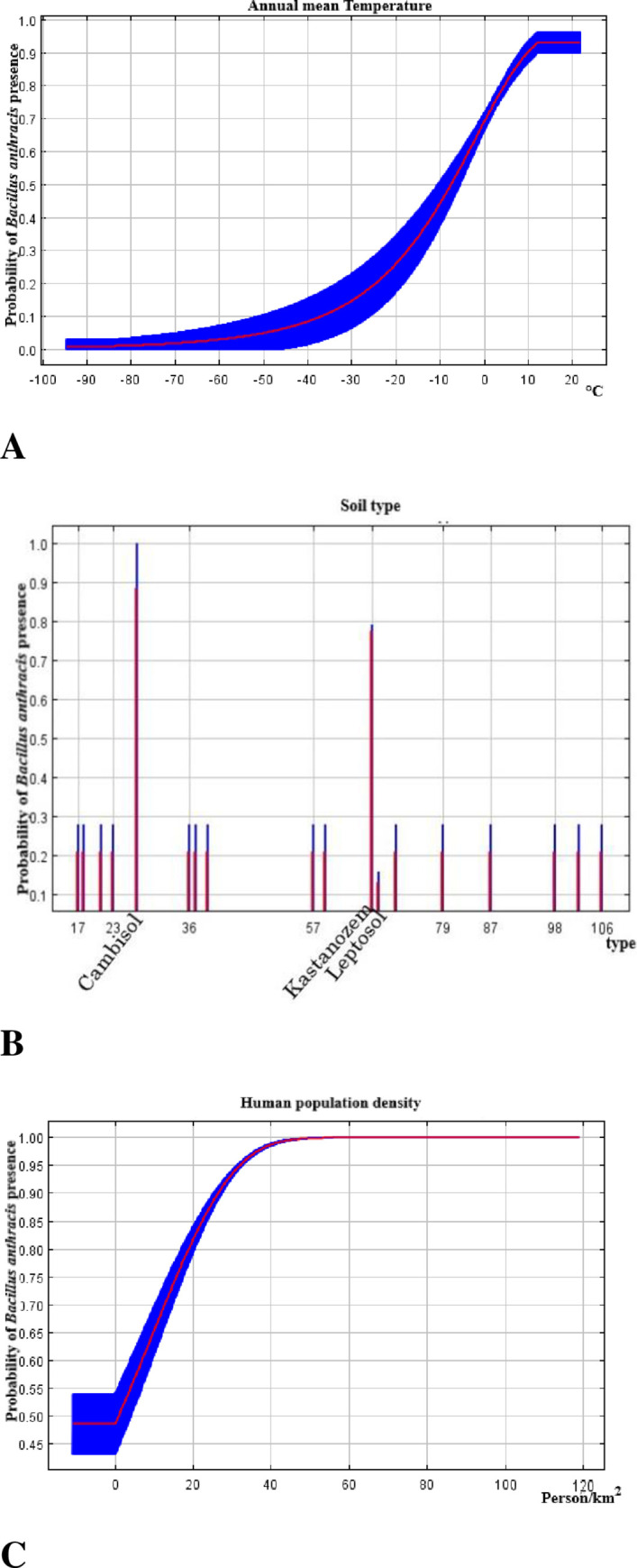
Response curves of continuous predictor variables (climate and human population density) and bar graph of categorical predictor variable (cover) for *B*. *anthracis* distribution in the Qinghai Lake basin. The red lines indicate the mean values while the blue lines denote the standard deviation.

**Fig 3:**
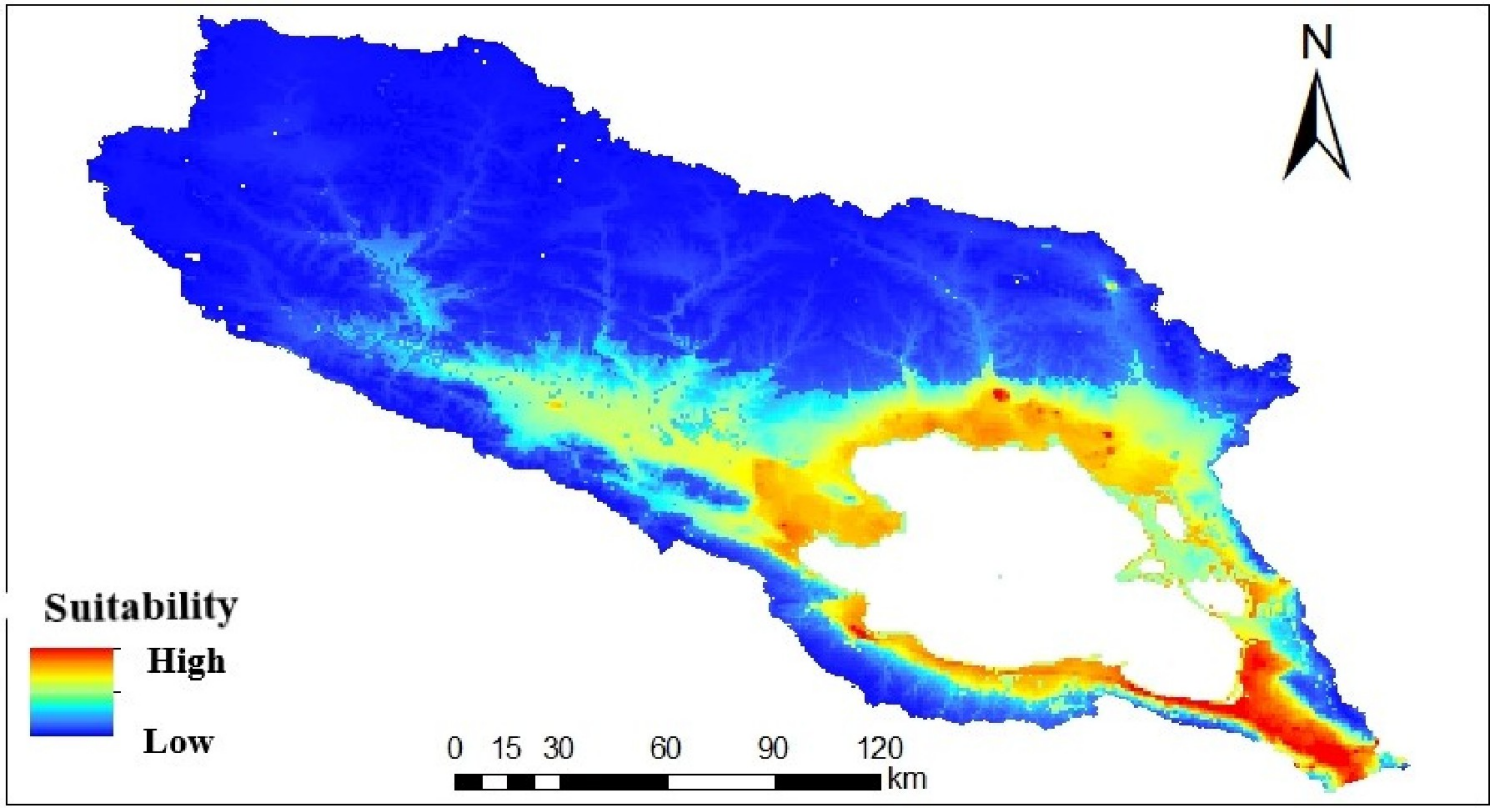
The environmental suitability map of *B*. *anthracis* distribution in the Qinghai Lake basin. Predictor variables used for modeling obtained from WorldClim (http://worldclim.org/version2), with kind permission of Dr. Stephen Fick, geo-spatial data scientist.

The suitability for the anthrax peaked when the annual mean temperature increased from -2 to 0°C, but declined briefly thereafter and maintained a constant probability across higher temperatures (Fig 2). The soil types with the highest suitability are cambisol and kastanozem, both in their Haplic subtype (Fig 2); Leptosols were unsuitable either in the presence of other variables or in isolation. The human population density response curve shows a gradual upward trend reaching a plateau at 40 individuals per km^2^ (Fig 2). Spatially, the highly suitable conditions are primarily found around Qinghai Lake. The northern and western part of the basin was predicted to be unsuitable for *B*. *anthracis* (Fig 3).

## Discussion

Our study presents the first assessment and spatial analysis of ecological suitability for *B*. *anthracis* in the Qinghai Lake basin. Although, epidemiological analysis had been done in Qinghai province [14] and anthrax distribution mapping in mainland China [15].

Here we analyzed over ten years mixed anthrax outbreaks using MaxEnt algorithms to investigate the environment and the geographic distribution of *B*. *anthracis* in the Qinghai Lake basin. The identified sets of environmental predictors of *B*. *anthracis* may represent factors directly or proxies thereof. This study supports the results of other studies, which have shown that anthrax outbreaks are associated with specific soils types [7], relatively high temperatures [19] and high human population density [15]. We found that relatively high mean annual temperatures within our high alpine environment had greatest predicted probability for anthrax occurrence. Consequently, continued climate warming may increase suitability for anthrax [8] also in our AOI.

Soil types and certain soil characteristics, such as high levels of organic matter, alkaline pH or calcium, were previously thought to facilitate the *B*. *anthracis* distribution [50, 51]. In our model, anthrax suitability was largely driven by two soil types, namely cambisols and kastanozems while leptosols showed the lowest *B*. *anthracis* suitability. The cambisols, occurs on young alluvial deposits in our AOI as well as worldwide. Cambisols are medium-textured and have a good structural stability, a high porosity, good water holding capacity and good internal drainage. Most cambisols have a neutral to weak acidity, a satisfactory chemical fertility and an active soil fauna [52]. The emergence of humus-rich kastanozems as the second most suitable soil type with the lowest standard deviations may be due to the presence of calcite (carbonate mineral) in its subsurface [50]. However, soil organic carbon, pH, cation exchange capacity, silt content and calcium were not predictive for *B*. *anthracis* distribution in our AOI. We found that the human population density was associated with anthrax which may be caused by the increased in human population. However, sheep, goat, and cattle population density both contributed to the model during the initial modeling but failed to meet the backward stepwise variable elimination criterion in our variable selection mode. The increase in human population density, settlement expansion, and seasonal migration would enhance human, livestock, and wildlife contact which would provide opportunity for *B*. *anthracis* transmission. The pastoralist nature of the population in Qinghai Lake basin could establish a human–animal interface either in pasture or in corrals. They also practice mixed farming; rearing animals and crop cultivation (mostly oilseed rape and highland barley) which are often used as fodder, while tillage would transpose dormant spores to the soil surface which increases anthrax emergence rate.

In research carried out in Gangcha county (northwest of the lake), the migration of farmers and livestock were assessed that follow a regulation moving between low land and high mountains. The pastures are divided into spring pastures, spring and winter pastures, autumn pastures and summer pastures arranged from south to north. The spring, autumn, spring and winter, and winter pastures have an average altitude of 3190 m, 3890 m, 3890 m, and 4015 m respectively. Sheep, cattle and yak are the most dominant livestock in our AOI, Sheep herding practices include high-altitude summer pasturing which may reduce exposure to *B*. *anthracis* at the high anthrax risk zone around the lake during summer months. Although, human population density may act in proxy but there are other factors for *B*. *anthracis* risk analysis and assessment such as: type of animal husbandry, the number and density of livestock herds per household, transhumance, and carcasses disposal methods. The later, if not well practice would create ‘locally infectious zones’ (LIZs) at carcass sites [53], and establish a demography of their own as these zones appear and fade over time. Rather than passively acting as a fomite, evidence suggests that anthrax carcass sites have a complex set of biotic interactions that determine their persistence and infectiousness within the area [11]. Other important factors such as the difference in the quality of veterinary surveillance, and anti-epidemic measures during an outbreak of anthrax, ulcers through slaughtering of sick and suspicious livestock, lack of preventive therapy among the rest of the livestock and people working with it could increase frequency of outbreak.

The areas with the highest suitability ranking are the low-lying area around the lake. The suitability could be dependent on the alluvial deposits, the various drainage channels from the higher elevation, characterized by the relocation of *B*. *anthracis* with soil through water, flooding or rain [50]. The result of our model with low altitude (around 3200 m.a.s.l) as an essential condition for survival of anthrax is in agreement with most similar studies on anthrax in South Africa [54], Zimbabwe [19] and Canada [55]. Our study reveals that bioclimatic and edaphic factors are fundamental conditions for *B*. *anthracis* distribution. Also, human population density and other related activities are specific factors reshaping the spatial distribution of *B*. *anthracis*.

Our results should be interpreted with the following limitations in mind. First, human and livestock cases could have been under-reported as the surveillance was passive. The size of the occurrence data could be associated with sampling bias such as reporting cases only at where there is higher population density rather than truly being absent [46]. Broadly, MaxEnt can perform well with small sample sizes [46]. High success rates and statistical significance has been observed in jackknife tests with sample size as low as five [56] and ten using MaxEnt algorithm [57]. The changes in the diagnostic criteria for human anthrax cases since 2008 might have affected the quantity of the reported data. Second, some risk factors were not available to enrich our model, including but not limited to some soil characteristics (organic matter, alkaline pH, calcium etc.), seroprevalence in human and livestock exposure level of people at risk, and the industrialization level of livestock production. These factors may have been influenced by the sample size and spatial resolution of available predictor variables.

## Conclusion

We categorized the Qinghai Lake basin into two suitability classes for *B*. *anthracis* distribution i.e., high and low, and revealed that increase annual mean temperature, two specific soil types (cambisols and kastanozems), and a high human population density, were the contributing variables for predicting *B*. *anthracis* environmental suitability. Soil type was the only significant categorical variables and second most influential variable overall; this would strengthen the edaphic paradigm for *B*. *anthracis* in its role for global *B*. *anthracis* suitability and anthrax epidemiology studies. Additionally, disease surveillance, health education, safe disposal of infected animal carcasses, vaccination of livestock, and other anthrax control measure strategies would be essential for disease prevention and can be prioritized for high-risk regions identified in our work.

## Supporting information

S1 Table Bioclimatic, elevation and classical meteorological variables used for initial modeling in MaxEnt software (T-Temperature and P–Precipitation) R. (DOC)

S2 Table Edaphic and other factors used in modeling. (DOC)

S3 Table Environmental variables used for the final MaxEnt model. (DOC)

S4 Table The record of anthrax outbreak with latitude and longitude information of the location. (DOC)

S1 Fig Scree plot of climate predictor variables showing a steep decline of eigenvalues across the component numbers. (TIF)

S2 Fig Average ROC and related area under the curve (AUC) of the selected model. (TIF)

## Abbreviation

MaxEnt: Maximum entropy
ESRI: Environmental systems research institute
GIS: Geographic information system
AUC: Area under the curve
ROC: Receiver operating characteristics
ISRIC: International soil reference and information
USDA: United state department of agriculture
SPSS: Statistical package for social sciences
PCA: Principal component analysis
AOI: Area of Interest
QLB: Qinghai Lake basin
