# Supplementary material for: Modeling the environmental suitability for Bacillus anthracis in the Qinghai Lake Basin, China
Source: PLoS One. 2022 Oct 14;17(10):e0275261. doi: 10.1371/journal.pone.0275261 (PMC9565420; doi:10.1371/journal.pone.0275261)
Supplement: S1 Table — (DOC) [file pone.0275261.s001.doc]

**S1 Table:** **Bioclimatic, elevation and classical meteorological variables used for initial modeling in maxent software (T- Temperature and P – Precipitation).**

| **Label** | **Variable description** | **Units** |
| --- | --- | --- |
| **1.     Bioclimatic variables** | | |
| Bio1 | Annual Mean T | °C |
| Bio2 | Mean Monthly Diurnal Range (Tmax - Tmin) | °C |
| Bio3 | Isothermally (BIO2/BIO7) x 100 | Index |
| Bio4 | T Seasonality (Standard Deviation) | °C |
| Bio5 | Max T of Warmest Month | °C |
| Bio6 | Min T of Coldest Month | °C |
| Bio7 | T Annual Range (BIO5-BIO6) | °C |
| Bio8 | Mean T of Wettest Quarter | °C |
| Bio9 | Mean T of Driest Quarter | °C |
| Bio10 | Mean T of Warmest Quarter | °C |
| Bio11 | Mean T of Coldest Quarter | °C |
| Bio12 | Annual P | mm |
| Bio13 | P of Wettest Month | mm |
| Bio14 | P of Driest Month | mm |
| Bio15 | P Seasonality (Coefficient of Variation) | Fraction |
| Bio16 | P of Wettest Quarter | mm |
| Bio17 | P of Driest Quarter | mm |
| Bio18 | P of Warmest Quarter | mm |
| Bio19 | P of Coldest Quarter | mm |
| **2.     Elevation** | | |
| Alt | Elevation | m a.s.l. |
| **3.     Classical meteorological variables** | | |
| Monthly P | Monthly P (n=12) | mm |
| Monthly  Tmean, Tmin, Tmax | Monthly mean, minimum and maximum T (n=36) | °C |
| ISR 1-12 | Average monthly Incoming Solar Radiation (n=12) | kJ m^-2^ day^-1^ |
