# Supplementary material for: Modeling the environmental suitability for Bacillus anthracis in the Qinghai Lake Basin, China
Source: PLoS One. 2022 Oct 14;17(10):e0275261. doi: 10.1371/journal.pone.0275261 (PMC9565420; doi:10.1371/journal.pone.0275261)
Supplement: S2 Table — (DOC) [file pone.0275261.s002.doc]

**S2 Table**: **Edaphic and other factors used in modeling.**

| **Variable** | **Value/Categories/Units** |
| --- | --- |
| Soil Types | categorical |
| Soil pH | 6.6-8.6 |
| Cation Exchange Capacity (CEC) | 0.5-6.1 meq/100 g |
| Silt content | 2.4-4.7 mg mL^−1^ |
| Calcium | 0-1.7 mmol/L |
| Soil Organic Carbon (SOC) | 0-18.1(lbs/ac) |
| Land use and cover | categorical |
