# Supplementary material for: Modeling the environmental suitability for Bacillus anthracis in the Qinghai Lake Basin, China
Source: PLoS One. 2022 Oct 14;17(10):e0275261. doi: 10.1371/journal.pone.0275261 (PMC9565420; doi:10.1371/journal.pone.0275261)
Supplement: S3 Table — (DOC) [file pone.0275261.s003.doc]

**S3 Table:** **Environmental variables used for the final maxent model.**

| **Variables** | **Type** | Value/Categories/Unit | Source |
| --- | --- | --- | --- |
| Annual mean T (Bio01) | Continuous | -9.7 to 1.3°C | WorldClim^a^ |
| Mean T of coldest Quarter (Bio11) | Continuous | -20.8 to -10.3°C | WorldClim^a^ |
| Elevation | Continuous | 3160 to 4921 m.a.s.l | WorldClim^a^ |
| Sheep population Density | Continuous | 0 to 861.99 individual/km^2^ | GLW3^b^ |
| Human Population Density | Continuous | 0 to 104.32 persons/km^2^ | WorldPop^c^ |
| Soil Type | Categorical | Haplic Arenosols (17), Haplic Arenosols (Calcaric) (18), Haplic Calcisols (21), Luvic Calcisols (23), Haplic Cambisols (27), Calcic Chernozems (36), Haplic Chernozems (37), Haplic Cryosols (39), Haplic Gleysols (54), Mollic Gleysols (57), Calcic Gypsisols (59), Haplic Gypsisols (60), Calcic Kastanozems (66), Haplic Kastanozems (67), Haplic Leptosols (68), Mollic Leptosols (71), Rendzic Leptosols (72), Haplic Luvisols (79), Haplic Phaeozems (87), Haplic Podzols (98), Aric Regosols (99), Haplic Regosols (Eutric) (102), Haplic Solonchaks (106). | Soilgrids^d^ |
| Land use | Categorical | Cropland (10), rice paddy (11), forest (20), Grassland (30), shrubland (40), permanent snow and ice (100), cloud (120), Grassland (130), sparce vegetation (fc < 0.15) (150), wetlands (180), impervious (190), bare area (200), unconsolidated bare area (202), water body (210), permanent ice and snow (220). | Soilgrids^d^ |

^a^T – Temperature; sources: Worldclim 1.4 (https://[worldclim.org](http://www.worldclim.org/)) at 30 arcsecond resolution.

^b^Source: Gridded Livestock of the World (https://livestockdata.org/contributor/gridded-livestock-world-glw3).

^c^Source: WorldPop Dataverse Repository (<http://worldpop.org.uk/data/>).

^d^Source: Soilgrids – global gridded soil information (http://www.isric.org/explore/soilgrids)
