# Supplementary material for: Modeling the environmental suitability for Bacillus anthracis in the Qinghai Lake Basin, China
Source: PLoS One. 2022 Oct 14;17(10):e0275261. doi: 10.1371/journal.pone.0275261 (PMC9565420; doi:10.1371/journal.pone.0275261)
Supplement: S4 Table — (DOC) [file pone.0275261.s004.doc]

**S4 Table: The record of anthrax outbreak with latitude and longitude information of the location.**

| Species | Longitude | Latitude |
| --- | --- | --- |
| Anthrax | 99.023 | 37.3009 |
| Anthrax | 100.64 | 37.08 |
| Anthrax | 100.849 | 36.844 |
| Anthrax | 100.582 | 37.296 |
| Anthrax | 100.994 | 36.402 |
| Anthrax | 99.715 | 36.765 |
| Anthrax | 102.015 | 35.5195 |
| Anthrax | 102.104 | 36.502 |
| Anthrax | 101.1 | 37.68 |
| Anthrax | 100.239 | 34.4774 |
| Anthrax | 99.9009 | 33.9692 |
| Anthrax | 98.8606 | 35.1014 |
| Anthrax | 101.62 | 37.37 |
| Anthrax | 97.0919 | 33.0117 |
| Anthrax | 100.146 | 37.3255 |
| Anthrax | 101.76 | 36.63 |
| Anthrax | 101.616 | 34.7348 |
| Anthrax | 100.62 | 36.2841 |
| Anthrax | 100.748 | 35.5867 |
| Anthrax | 99.403 | 37.157 |
| Anthrax | 99.882 | 37.303 |
| Anthrax | 100.821 | 36.559 |
| Anthrax | 100.299 | 36.621 |
| Anthrax | 98.13 | 34.55 |
| Anthrax | 99.48 | 35.05 |
