# Supplementary figures and images for: Modeling the environmental suitability for Bacillus anthracis in the Qinghai Lake Basin, China
Source: PLoS One. 2022 Oct 14;17(10):e0275261. doi: 10.1371/journal.pone.0275261 (PMC9565420; doi:10.1371/journal.pone.0275261)

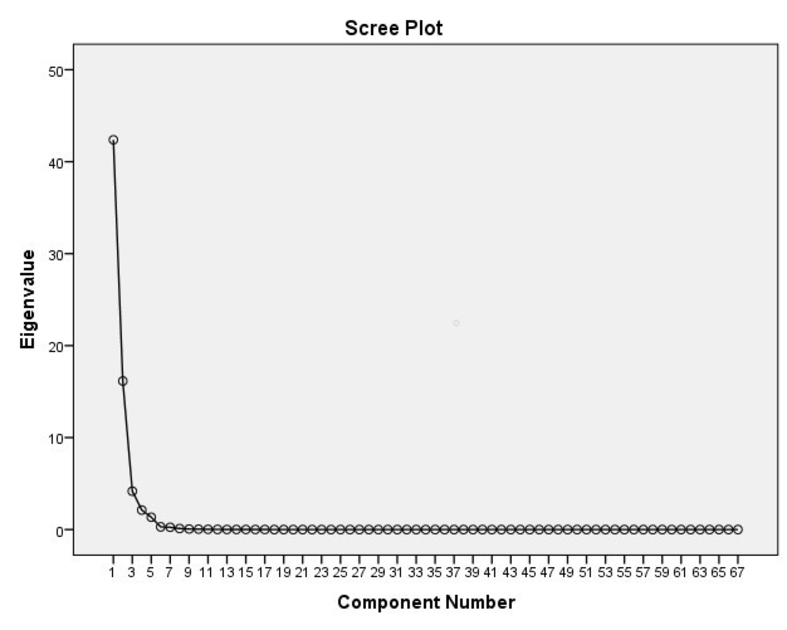

Supplement: S1 Fig — (TIF) [file pone.0275261.s005.tif]

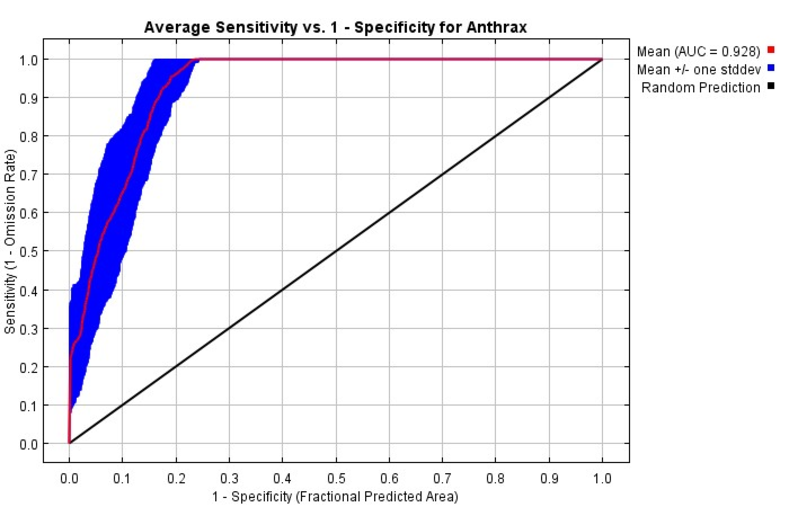

Supplement: S2 Fig — (TIF) [file pone.0275261.s006.tif]
